# Review of the clinical electrooculogram - Part 2: the bestrophinopathies and modified protocols

**DOI:** 10.1007/s10633-026-10093-y

**Published:** 2026-03-08

**Authors:** Srikanta Kumar Padhy, Maja Šuštar Habjan, Paul A. Constable

**Affiliations:** 1 Anant Bajaj Retina Institute, LV Prasad Eye Institute, Mithu Tulsi Chanrai Campus, Bhubaneswar, 751024 India; 2 Department of Ophthalmology, University Medical Centre Ljubljana, Grabloviceva 46, 1000 Ljubljana, Slovenia; 3 College of Nursing and Health Sciences, Caring Futures Institute, Flinders University, Adelaide, Australia

**Keywords:** Bestrophin, Light-rise, Retinal pigment epithelium, Protocol, Standing potential, Maculopathy

## Abstract

The light-rise of the electro-oculogram (EOG) is used as a clinical marker for a collection of disorders known as the ‘bestrophinopathies.’ This review provides an overview of these conditions including Best Vitelliform Macular Dystrophy (BVMD, Autosomal Recessive Bestrophinopathy (ARB), Adult Onset Vitelliform Macular Dystrophy (AVMD) and Autosomal Dominant Vitreoretinalchoriodopathy (ADVIRC) and potential future therapies. One drawback of the EOG is the time to administer the test and shortened protocols that have been developed to improve the clinical testing of the EOG which include incorporating measures during recordings of the ERG or shortening the period of dark and light adaptation. The companion paper summarizes the cellular mechanism of the EOG, and this review is focused on the clinical applications of the EOG.

**Supplementary Information:**

The online version contains supplementary material available at 10.1007/s10633-026-10093-y.

## Background

The clinical electrooculogram (EOG) [1] is a functional test of the retinal pigment epithelium (RPE) that supports visual processes of the outer retina [2]. The RPE forms a monolayer of cuboidal cells that are hexagonally arranged with the highest density at the fovea [3]. The apical membrane has microvilli that interdigitate with the outer segments of the rod and cone photoreceptors. The basolateral membrane is continuous with the underlying Bruch’s membrane [4]. Tight junctions are formed between the RPE cells to form a selective barrier and provide electrical resistance between the apical and basolateral membrane electrical potentials [5]. The functions of the RPE are diverse including fluid regulation, light absorption, phagocytosis, ionic homeostasis, glucose transport to the photoreceptors and growth factor secretion to maintain permeability of the underlying choroid and maintain photoreceptor function [2]. The clinical EOG is helpful in conditions that affect the role of bestrophin primarily in diagnosing a collection of disorders referred to as ‘the bestrophinopathies [6, 7] arising from pathogenic variants in *BEST1* [8]. This paper focusses on the clinical aspects of the EOG whilst the accompanying companion paper explores the current mechanism of the light-rise of the EOG with particular emphasis on the role of bestrophin in regulating intracellular calcium and basolateral chloride conductance through voltage gated anoctamin channels.

The bestrophinopathies include a spectrum of retinal disorders caused by disease-causing variants in the *BEST1* gene. These include Best vitelliform macular dystrophy (BVMD), adult-onset vitelliform macular degeneration (AVMD), autosomal dominant vitreoretinochoroidopathy (ADVIRC), and autosomal recessive bestrophinopathy (ARB) [7, 9]. These conditions primarily affect the macula and are typically present within the first two decades of life. *BEST1* pathogenic variants affect trafficking and normal functioning of the L-type Ca^2+^ channel including phagocytosis [10, 11], cell volume regulation [12] further contributing to the pathophysiology of the bestrophinopathies [13, 14]. The macular vitelliform lesions that characterize BVMD may be a result of the low expression of bestrophin in this region of the eye making the central region more susceptible to pathological changes [15]. Despite its milder phenotype, AVMD remains clinically indistinguishable from early-stage BVMD in many cases. This phenotypic overlap has led to the proposal that AVMD represents either an attenuated [16] or a sub-type of BVMD [17] rather than a distinct entity. It should be noted that although *BEST1* variants are the most widely recognized cause of vitelliform lesions, adult-onset presentations are genetically heterogeneous, with *PRPH2* [18, 19], *IMPG1, IMPG2,* [20–22], *HTRA1* [23], and *CTNNA1* [24], with others also implicated. *PRPH2* being most common and *BEST1/VMD2* the second most common [18].

The EOG is typically abnormal in conditions affecting the outer retina/RPE or the choroid such as secondary to drug toxicity or ischemia. Conditions in which abnormal EOGs, characterized by a reduction in the light-rise have been reported include pigmented paravenous retinochoroidal atrophy [25], posterior multifocal placoid pigment epitheliopathy [26], pattern dystrophy [27], X-linked carriers of retinitis pigmentosa [28], membranoproliferative glomerulonephritis type II [29], gyrate atrophy [30], Helicoidal peripapillary chorioretinal degeneration [31], unilateral acute idiopathic maculopathy [32] and North Carolina Macular Dystrophy (NCMD) [33]. Whilst the main use of the EOG is in the bestrophinopathies, other clinical conditions may reveal reductions in the light-peak such as Danon’s Disease with low Dark Trough (DT) and LP amplitudes [34] and bilateral optic neuropathy [35]. Vigabatrin therapy can lead to loss of visual fields and reduced EOGs secondary with a loss in RPE function [36]. The EOG may be affected in chloroquine retinopathy but is not recommended as a standard screening tool [37]. A case report following tamoxifen retinopathy has been reported with a reduced LP:DT_ratio_ [38]. Most commonly the EOG is associated with a collection of disorders associated with pathogenic variants in *BEST1* [39] that result in visual loss and are associated with the accumulation of exudate in the sub retinal space [40]; with outer retinal changes associated with worse visual acuity [41]. The spectrum of phenotypes relating to BVMD, ARB and ADVIRC depend upon the pathogenic variants in *BEST1* [42]. Large phenotype-genotype descriptions have been described for BVMD in Chinese [43], Italian [44], United Kingdom [45, 46], and Danish [47] populations.

The EOG is of benefit when diagnosing conditions affecting bestrophin, the clinical test can be arduous and time-consuming in the clinic. Shortened protocols that integrate the EOG recording into the ISCEV standard ERG recording protocol have been proposed [48]. In addition, reducing the dark- and light-adapted times to 6- and 10-min intervals respectively to reduce the overall EOG test time [49]. Modifying the existing ISCEV standard test protocol [1] may help to improve the clinical utility of the EOG as a test for RPE function. For a review of the current EOG recording protocols and mechanism see the companion paper and Constable (2024) [50], Constable (2014) [51] and Arden and Constable (2006) [52].

## Bestrophinopathies

Disease causing variants in *BEST1* cause mislocalization or loss of function, but the specific pathogenic variant is not related directly to the disease phenotype [11]. The compound heterozygous R141H and Y29stop pathogenic variants were identified in a Swedish family with BVMD [53] with another early report identifying a variant in in exon 8 p.E292K with all affected individuals having reduced light-peak to dark-trough ratios (LP:DT_ratios_) [54]. When BVMD disease causing variants associated with *BEST1* were explored including (V9M, W93C, and R218C) in transfected Madin-Darby Canine Kidney (MDCK) cells the expression profile revealed that the R218C variant in Best1 was basolaterally localized with the W93C and V9M Best1 variants deposited intracellularly. The authors concluded that there were likely to be at least three basolateral sorting motifs that affected the localization of bestrophin [55]. Localization has also been implicated in the following pathogenic variants following transfection in cell lines (Y85H, Q96R, L100R, Y227N and Y227E) [56], and (T6P, L21V, W24C, L224M, Y227N, T237R, F305S and V311G) also show trafficking errors with these disease causing variants on the cytoplasmic face compared to pathogenic variants in the membrane domain (S79C, F80L, L82V and A243T) [57].

Whilst no treatment is currently available, gene therapies are being explored that may prevent visual loss through the restoration of *BEST1* function [58–63]. Therapeutics targeting *BEST1* also show promise with 25 μM tadalafil restoring bestrophin function *in-vitro* [64], 4-phenylbutyrate (4PBA) also restoring function in transfected cell lines [65] and more recently in RPE cells derived from patients with dominant and recessive bestrophinopathies [66]. A natural substance, curcumin, has also increased bestrophin function and tight junctions in iPSC-RPE cells [67]. An early clinical trial using docosahexaenoic acid supplementation showed no clinical efficacy [68].

Human embryo-derived pluripotent stem cells (PSC-RPE) [69] and induced pluripotent stem cells (iPSC-RPE) [70, 71] cultures provide a new method to explore therapeutic and gene-based therapies *in-vivo* [59, 72–75]. Gain-of-Function (GOF) through gene editing has also been demonstrated in *in-vivo* with increased TMEM-16A currents [76]. Transfection in human RPE cell cultures with plasmids carrying three *BEST1* disease causing variants p.V143F, p.S142G, and p.A146T initiated apoptosis of RPE cells [70]. Successful restoration of retinal structure has been demonstrated with gene therapy in canine that holds promise for translation to humans with canine disease exhibiting similar characteristics to human BVMD [77].

Disease-causing variants in the *peripherin/RDS* gene are a recognized cause of autosomal dominant macular dystrophies, including AVMD, pattern dystrophies, and cone-rod degeneration. Most pathogenic variants are missense variants in the intradiscal D2 loop, a region essential for protein folding and ROM-1 interaction. The P210R pathogenic variant is one of the most studied, showing intrafamilial variability and a wide phenotypic spectrum from vitelliform lesions to geographic atrophy and choroidal neovascularization [78]. Detection rates vary across populations, from 7 to 23%, and frameshift variants are also reported in certain phenotypes like multifocal pattern dystrophy. Although *peripherin/RDS* is important, it accounts for only a minority of AVMD cases, highlighting the genetic heterogeneity of adult-onset macular dystrophies. Genotype–phenotype correlations suggest missense pathogenic variants may lead to severe disease, while Loss-of-Function (LOF) variants are linked to milder forms [79].

## Best’s vitelliform macular dystrophy

Best vitelliform macular dystrophy (BVMD) is an autosomal dominant macular dystrophy caused by pathogenic variants in *BEST1*, encoding the calcium-activated chloride channel Bestrophin-1 that is localized to the endoplasmic reticulum and basolateral membrane of the retinal pigment epithelium (RPE) [80, 81] at the basolateral RPE. It typically begins in childhood and is characterized by abnormal RPE–photoreceptor interaction and reduced EOG light-rise with a generally normal ffERG until late disease [9, 53, 82].

BVMD progresses through defined clinical stages: an early pre-vitelliform stage with subtle Optical Coherence Tomography (OCT) abnormalities and possible LP:DT_ratio_ reduction; the classic vitelliform (“egg-yolk”) stage with subretinal accumulation of lipofuscin-rich material; a pseudohypopyon stage where material layers inferiorly; a vitelliruptive or “scrambled-egg” stage marked by fragmentation and resorption; and a final atrophic/fibrotic stage with photoreceptor loss, macular thinning, and potential choroidal neovascularization (CNV) [45, 46]. Although this sequence is typical [40], disease severity and progression rate vary widely [9, 45, 46, 83], and atypical presentations-including unilateral involvement [84], diabetic retinopathy [85], focal choroidal excavation [86], macular hole [87], and CNV-have been reported [88].

**Fig. 1:**
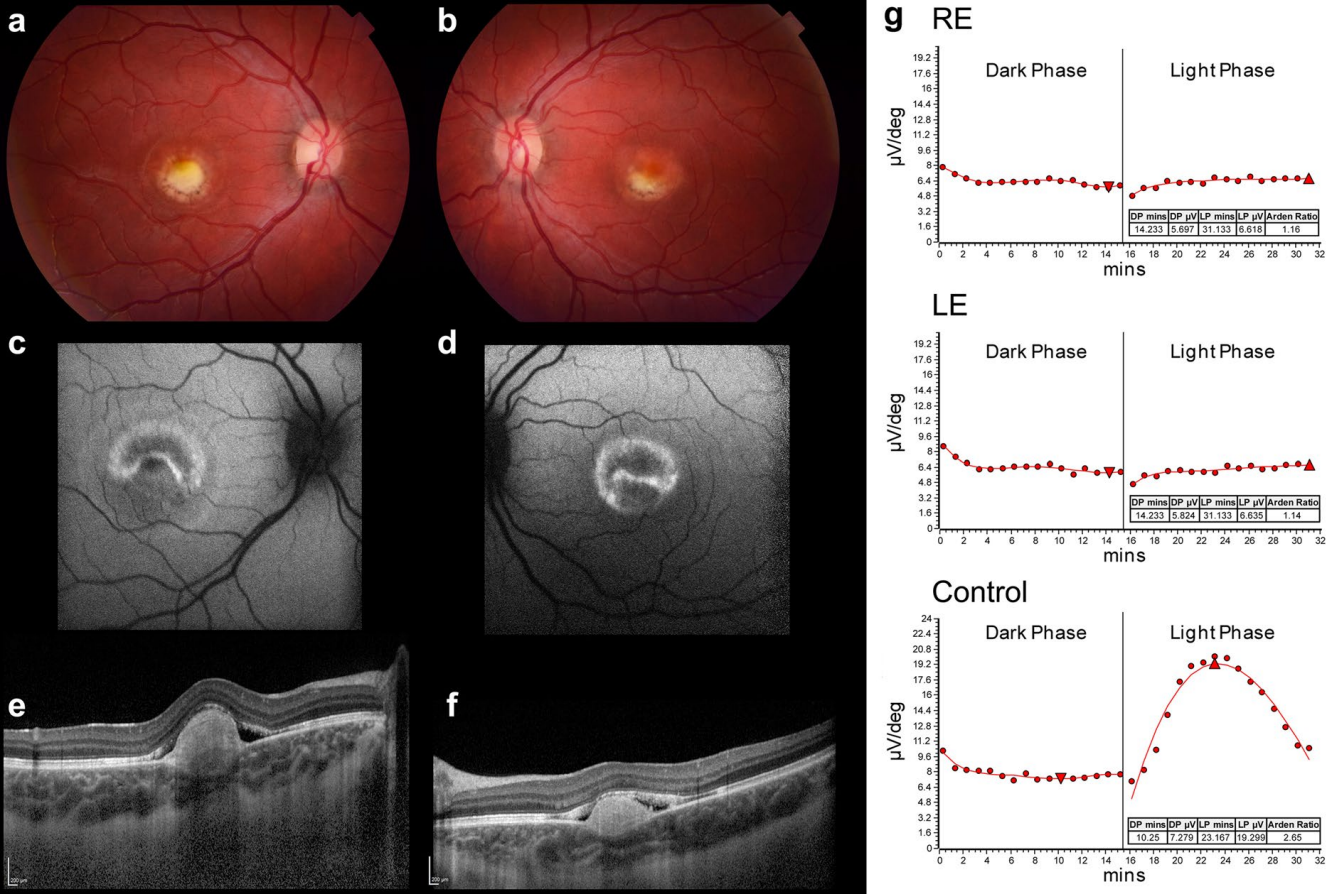
Typical clinical presentation of Best Vitelliform Macular Degeneration. Color fundus photographs **(a, b)** showing central vitelliform with hyper and hypo fluorescent contents containing fluid and lipofuscin **(c, d)**. Optical Coherence Scan through the macula showing the bilateral raised central lesions **(e/f)**. Electrooculograms of the Right (RE) and Left eyes (LE) show a flat response to a change in the standing potential during the light phase. In contrast the Control electrooculogram exhibits a light-rise during the light phase with an increase in the standing potential that is dependent on normal bestrophin function within the retinal pigment epithelium

Early changes in BVMD affect the interdigitation between the outer segments and apical membrane of the RPE [89] and have been observed in individuals less than 10 years of age [90]. Pre-clinical investigations in six individuals with *BEST1* disease causing variants reported a decreased LP:DT_ratio_ in 5 of 12 eyes and abnormal OCT findings in three subjects with p.V9A; p.R92C; p.I230T variants characterized by heightened reflectance between the RPE and outer segments [91]. Quantitative fundus autofluorescence is reported as normal except for the intense fluorescence associated with the bullous central detachment containing lipofuscin [92] that contains increased accumulation of bis-retinoid N-retinyl-N-retinylidene ethanolamine (A2E) [93]. Reduced global chromatic and achromatic contrast sensitivity reduces with disease duration [94]. The vitelliform lesion, the hall mark of BVMD typically is described as having an ‘egg-yolk’ appearance of accumulated fluid and lipofuscin [95]. Imaging studies using near infrared autofluorescence may be helpful in determining the pre-vitelliform stage [96] with 34.7% showing regions of fundus autofluorescence at the posterior pole and a reduction in central retinal thickness with age [46]. Case reports have reported unusual presentations including bilateral macular holes [97], unilaterally [82, 84], focal choroidal excavations [98], rarely a near normal LP:DT_ratio_ [99], bulls-eye maculopathy [100], peripheral retinal schisis [101], and choroidal neovascularization [88]. Clinicians should be aware that similar findings to BVMD have been reported in NCMD [33] and that, an abnormal EOG with a normal ERG, long considered characteristic of Best vitelliform macular dystrophy (BVMD), is not unique to BEST1-related disease. Small et al. (2022) [33] demonstrated that patients with NCMD can exhibit the same electrophysiologic profile, challenging the specificity of the EOG as a diagnostic marker and indicating that reduced EOG light rise may reflect localized rather than widespread RPE dysfunction. This phenotypic overlap reinforces the need for molecular confirmation and multimodal assessment, particularly in the context of patient selection for emerging *BEST1*-targeted gene therapies. See Bianco et al. (2024) [83] for a review of the clinical features of BVMD and Fig. 1.

Disease causing variants in *BEST1* include a heterozygous c.614 T > C (p.I205T) pathogenic variant in exon 5 [102], in a Chinese population novel disease-causing variants include (T2N, L75F, S144N, R255W, P297T, D301G and R218C) [103]. Dominant bestrophinopathies are predominantly associated with missense variants located in functional regions of *BEST1* with clinical features including hyperopia and glaucoma overlapping with ARB [43].

Clinical electrophysiology in BVMD typically demonstrates a reduced to absent EOG LP:DT_ratio_—with reported values ranging from an absent light-rise to a LP:DT ratio of 3.10 [9, 18, 20, 21, 47, 53, 54, 82, 84, 85, 100, 101, 104, 105] (see Table S1 in Supplementary Material). The ffERG findings are generally normal in early disease stages under both dark- and light-adapted conditions. However, as BVMD progresses, delays in peak times and reductions in amplitudes may develop [106, 107]. Macular function assessed using mfERG demonstrates reduced amplitude in the central two rings, which correlates with the degree of subretinal fluid [47]. Polosa et al. (2025) [107] reported similar findings in adult patients. The pERG reveals reductions in p50 and n95 amplitudes when visual acuity falls below 0.5 LogMAR [106] or when photoreceptor loss occurs [108].

## Autosomal dominant vitreoretinochoroidopathy

ADVIRC (Autosomal Dominant Vitreoretinochoroidopathy) is a distinct *BEST1*-associated disorder characterized by a peripheral circumferential band of retinal hyperpigmentation, which is a consistent feature across all ages [109]. The disease is typically slowly progressive, with initial preservation of central vision and visual fields, but with gradual loss of peripheral field and retinal function over time [110]. First described by Kaufman et al. (1982) [109] in a family that exhibited peripheral hyperpigmentation, cataract, high myopia with loss of retinal function in advanced stages. Mainguy et al. (2024) [110] first reported the c.1101–1 G > T splice-site pathogenic variant in *BEST1* causing ADVIRC, demonstrating altered splicing and intrafamilial phenotypic variability. The ffERG findings are generally well preserved but have been reported to range from normal to severely reduced amplitudes, that correlate with age and disease severity, based on a retrospective review of a family containing 6 members with the p.V86M variant [111]. In a novel case report of a 33-year-old female who was heterozygous for c.1101–1 G > T variant resulting in an in-frame deletion (p(S367_N579del)) and exclusion of exon 10 with reduced scotopic ffERGs and diminished light-rise and bilateral hyperpigmented and atrophic hyperpigmented peripheral retina. Of note were that her mother and half-sister who were carriers of the same disease-causing variant also exhibited fundus changes similar to the presenting case [110]. The long-term changes in ADVIRC were reported in a case after twenty-years follow-up who developed macular atrophy and unilateral hyperpigmentation with the authors suggesting that chorioretinal atrophy of the macula can occur in the later stages of ADVIRC [112].

Importantly, ADVIRC is frequently associated with developmental ocular anomalies, including microcornea, angle-closure glaucoma, and cataracts [111, 113]. Associations such as iris dysgenesis and optic nerve dysplasia have also been reported. These anomalies, along with the retinal phenotype, may result from aberrant splicing of *BEST1* transcripts, leading to internally deleted or duplicated exons [110]. The commonly reported p.V86M pathogenic variant, for instance, has been linked to such splicing defects and abnormal EOG responses, although mild or early phenotypes may occasionally show a borderline light-rise [111]. For a review of pathogenic variants associated with BVMD the reader is directed to White et al. (2000) [114].

## Autosomal recessive bestrophinopathy

Autosomal recessive bestrophinopathy (ARB) commonly presents with multifocal vitelliform lesions and irregularities of the RPE, which appear as mixed areas of increased and decreased autofluorescence at the posterior pole [115]. Additional ocular findings frequently include intraretinal fluid [116], hyperopia, and, in certain individuals, shallow anterior chambers that increase the risk for angle-closure glaucoma [117]. ARB was first described by Burgess et al. (2008) [117] with a severely diminished or absent light-rise with reduced and delayed light- and dark-adapted ERGs and pattern ERGs. In a case report of a child a reduced LP:DT_ratio_ was described and mfERG reductions as well as delayed peak times for ERG, with associated RPE deposits and outer retinal changes with elongated and thickened photoreceptor outer segments, narrow anterior chambers has also been reported [118]. Modelling of ARB disease-causing variants suggest that the main reason for pathological changes is due to a lack of trafficking of bestrophin-1 [119].

Two novel pathogenic variants, c.202 T > C (chr11:61,722,628, p.Y68H) and c.867 + 97G > A, in *BEST1* have been reported in two ARB families using third generation sequencing [120]. Novel pathogenic variants also include compound heterozygous for (L40P) and (A195V) *BEST1* variant [121]. Other homozygous variants including, c.521_522del and c.1100 + 1G > A have also been reported [122]. Not all variants associated with ARB result in complete loss of function. Given the autosomal recessive inheritance pattern, a single pathogenic allele is not expected to cause disease in isolation. Accordingly, the R141H (CGC > CAC) variant alone (*Best1/Best1*^*R141H*^) does not produce a clinical phenotype and may represent a hypomorphic allele with partial residual function rather than a null variant. However, when R141H occurs in trans with the truncating mutation I366fsX18 (c.1098_1100 + 7del), the compound heterozygous state (*Best1*^*R141H*^*/Best1*^*I366fsX18*^) results in ARB, characterized by a depressed LP:DT_ratio_ and reduced visual acuity in the 14-year-old female patient [123]. Functional studies demonstrated that when this compound variant was expressed in Human Embryonic Kidney (HEK293) cells, whole-cell chloride currents were comparable to those observed in cells transfected with wild-type Best1, indicating that loss of bestrophin-1 anion channel activity is unlikely to be the primary mechanism underlying the pathophysiology of ARB in this case [123].

**Fig. 2:**
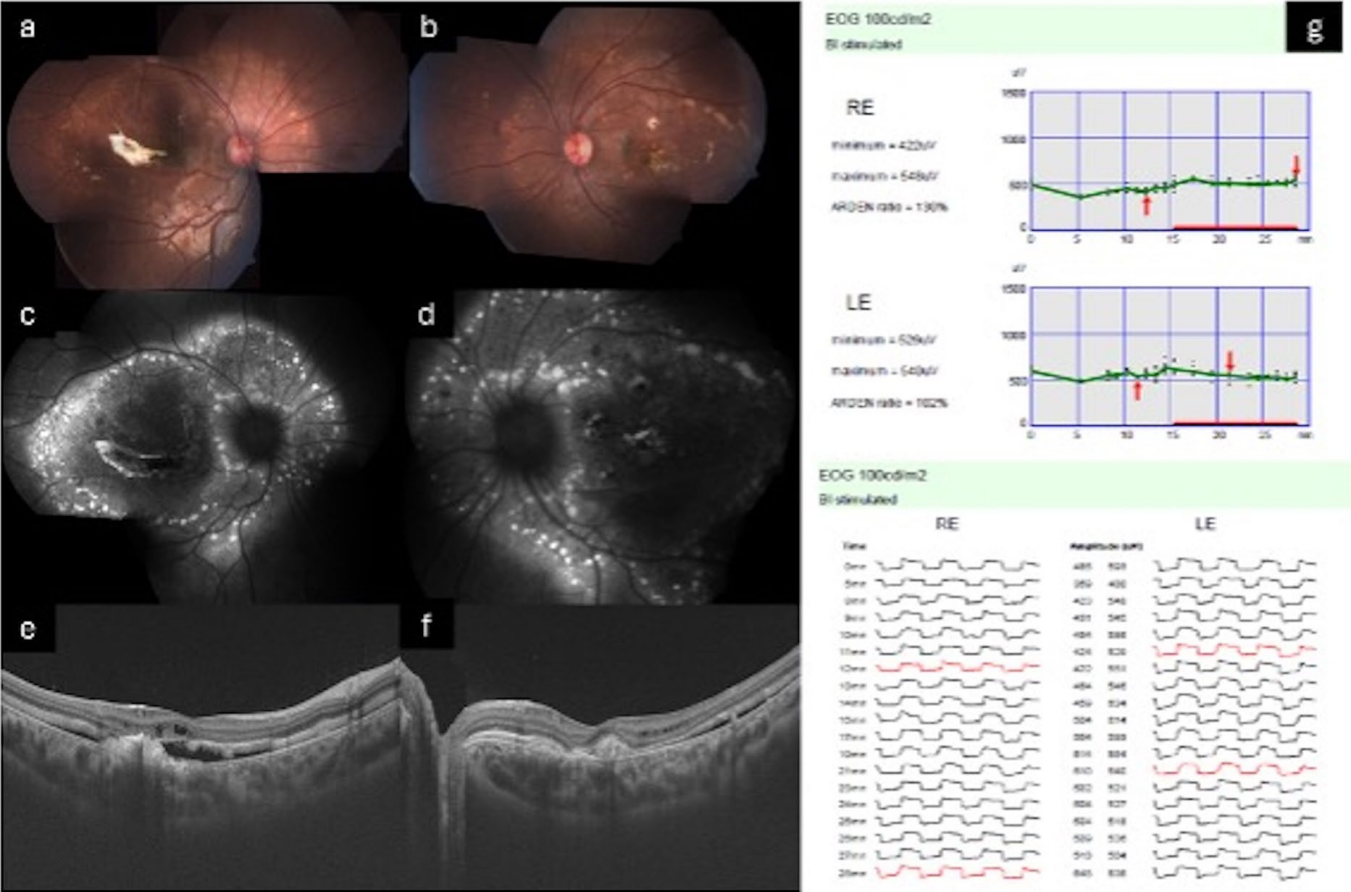
**a, b** Color fundus photographs showing multiple subretinal yellow punctate lesions along the vascular arcades, peripapillary region, and nasal to the optic disc, with evidence of a scarred choroidal neovascular membrane (CNVM). **c, d** Fundus autofluorescence images demonstrating discrete areas of hyperautofluorescence corresponding to the yellow deposits seen clinically. **e, f** Swept source OCT scans revealing a subretinal hyperreflective membrane, intraretinal schisis, and a subretinal hyporeflective space. **g** Electro-oculography of both eyes showing an absent light peak, with the LP:DT ‘Arden’ ratios of 130% in the right eye and 102% in the left eye

Clinically, EOGs reveal a severely reduced LP:DT_ratio_ in virtually all cases, irrespective of patient age, highlighting widespread RPE dysfunction. This abnormality is typically bilateral and symmetric. The degree of EOG reduction is disproportionate when compared to full-field ERG abnormalities, underlining the primary RPE involvement characteristic of ARB. The LP:DT_ratios_ have been reported to range between severely reduced [117] to 1.89 [118] with most studies reporting values of approximately 1.00 (See Supplementary Material Table S1). The most common ERG abnormalities include reduced amplitudes and delayed peak times across both dark-adapted and light-adapted responses. Younger patients often show milder or even normal ERG findings, which correlate with better visual acuity. In contrast, older patients typically exhibit more pronounced amplitude reductions and delayed implicit times. Longitudinal follow-up in some individuals confirms progressive decline, particularly in scotopic ERG components, while cone-driven responses tend to deteriorate more slowly [118, 119]. See Fig. 2 for clinical features of ARB.

## Adult onset vitelliform macular dystrophy

Adult onset Vitelliform Macular Dystrophy (AVMD), is often considered part of the spectrum of dominantly inherited bestrophinopathies and typically manifests between the ages of 30 and 50 years, with a slight female predominance [124]. Patients present with characteristic vitelliform lesions, usually smaller and less pronounced than those observed in BVMD. Although initially described as autosomal dominant, many sporadic AVMD cases likely reflect autosomal dominant inheritance with incomplete penetrance. Associated genes include *PRPH2*, *BEST1*, *IMPG1*, and *IMPG2* [125], although sporadic cases have been reported with negative genotypes for these genes [126].

Although AVMD shares a considerable phenotypic overlap with BVMD, it generally follows a milder course with later onset and slower progression [127]. Most patients maintain relatively preserved central vision, and progression through the classic stages of BVMD is not universal in AVMD. Nevertheless, complications such as macular atrophy and, less commonly, CNV or pigment epithelial detachment (PED) can occur [39].

Histopathological studies of AVMD are limited but have consistently demonstrated disruptions in the outer retina and RPE, with material accumulation in the subretinal space attributed to dysfunctional RPE metabolism. Reports highlight RPE hypertrophy, photoreceptor degeneration in the foveal region, pigment-laden macrophages, and the presence of periodic acid–Schiff (PAS)-positive material between the RPE and Bruch’s membrane. The role of lipofuscin accumulation remains debated, with variability across studies potentially reflecting disease stage at the time of tissue sampling [17, 128–131].

**Fig. 3:**
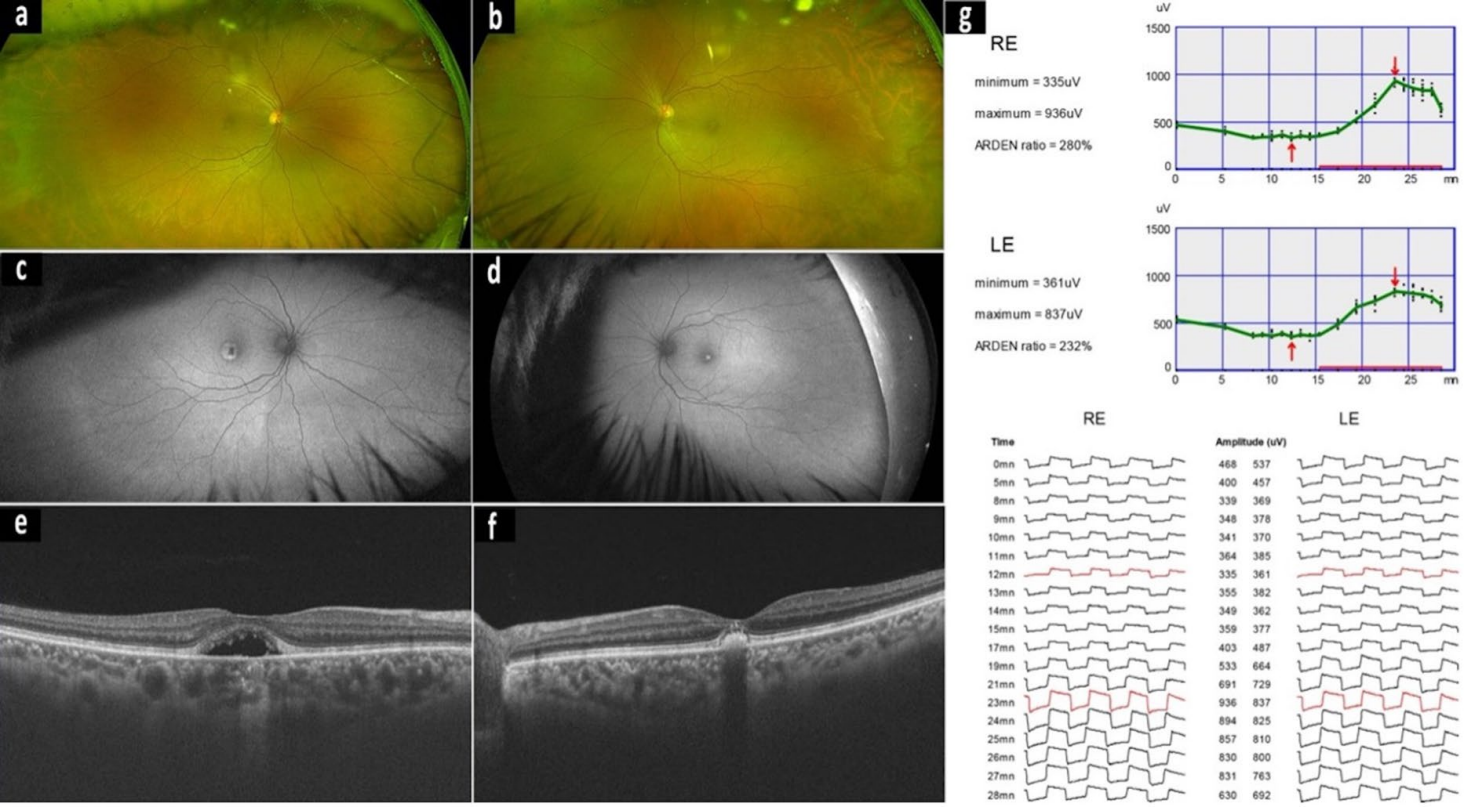
A case of a 45-year-old male with adult onset foveovitelliform dystrophy. Best corrected visual acuities were 6/6 both eyes. Optus wide field colour fundus photographs **a, b** of the right and left eyes. Fundus autofluorescence (**c, d)** indicates central hyper and hypofluosecence centred on the fovea. OCT reveals a disruption to the retinal pigment epithelium and fovea with a similar pattern to BVMD. The EOGs **(g)** are within normal limits for the LP:DT ‘Arden’ ratio with a light-rise

Electrophysiological testing in AVMD typically reveals preserved overall retinal function with localized macular dysfunction. The ffERGs indicate normal generalized retinal responses. In contrast, mfERG consistently demonstrates reduced responses from the central macula, correlating with the focal nature of the disease [132]. Electrooculography findings are variable but usually normal or only mildly reduced, with LP:DT_ratios_ often remaining within or just below normal limits [105]. Reported values for the LP:DT_ratio_ range from normal/reduced [18] to 3.04 [22] and reported range typically lie between 1.30–1.50 (See Supplementary Material Table S1). This contrasts with BVMD, where EOG is consistently abnormal due to widespread RPE dysfunction. The electrophysiological profile of AVMD—normal ffERG and EOG with abnormal mfERG- underscores the localized nature of the pathology and helps differentiate it from inherited bestrophinopathies such as BVMD. See Fig. 3 for clinical features of AVMD.

## Future therapies

Gene therapy is emerging as a promising approach for bestrophinopathies. The human eye presents an ideal target for these therapies due to its relative immune privilege, bilateral symmetry (enabling the use of the fellow eyes as a control), and anatomical accessibility for direct visualization and monitoring [133]. The *BEST1* gene is relatively small, and its expression is restricted to the RPE, making it well-suited for delivery using adeno-associated virus (AAV) vectors. Moreover, bestrophinopathies often exhibit a slow rate of progression, providing a generous therapeutic window [104]. However, this slow and gradual progression may also impede therapeutic development by complicating endpoint detection and extending trial duration. Structural hallmarks, such as subretinal vitelliform lesions or fluid, serve as quantifiable endpoints for assessing treatment response in early-phase clinical trials using OCT, potentially enabling smaller sample sizes and streamlined regulatory pathways [62].

## Preclinical evidence from animal models

Canine multifocal retinopathy (cmr), caused by naturally occurring biallelic *BEST1* pathogenic variants, closely mimics the clinical, histological, and molecular features of human ARB [134]. A study by Guziewicz et al. (2018) [135] showed that early disease in cmr is marked by micro detachments at the RPE–photoreceptor interface, linked to impaired calcium signalling and underdeveloped apical RPE microvilli. Subretinal injection of either canine or human *BEST1* using AAV vectors led to lesion reversal within 4–12 weeks and durable improvements up to 245 weeks post-injection without significant inflammation [135]. Notably, this gene replacement restored both microstructural architecture and functional adhesion at the RPE–photoreceptor junction, confirming the viability of this approach for early and advanced ARB.

In contrast, *BEST1*-knockout mice do not exhibit a phenotype, with a near normal normal light rise, limiting their utility [136]. However, knock-in mice harbouring the W93C disease-causing variant (seen in BVMD) demonstrate clinical features such as dominant inheritance, reduced EOG light-rise, and serous retinal detachments, making them valuable for studying dominant variants [137]. A spontaneous *BEST1* pathogenic variant *p.Q327E* has been identified in the primate *Macaca fascicularis* that could support studies into disease progression and therapeutic targets [138].

## Insights from iPSC-RPE models

Patient-derived induced pluripotent stem cells (iPSCs), differentiated into RPE, offer a powerful in vitro platform for modelling disease and testing therapy. These iPSC-RPEs recapitulate disease-specific defects in *BEST1*-mediated chloride conductance and permit pathogenic variant-specific assessments. Baculovirus-mediated supplementation has rescued Cl⁻ currents in ARB iPSC-RPEs [74]. Furthermore, dominant disease-causing variants-if not GOF-can be effectively rescued by overexpressing wild-type (WT) *BEST1*, supported by the tolerability of *BEST1* overexpression in canine models [59]. Notably, both recessive and dominant pathogenic variants with LOF or dominant-negative (DN) behaviour-but without toxic GOF effects-are amenable to gene augmentation therapy using AAV, lentiviral, or baculoviral vectors [59]. Restoration of chloride channel function is often dose- and time-dependent, regardless of inheritance pattern [76].

## Overcoming gain-of-function challenges

Some dominant *BEST1* disease-causing variants exert a DN effect via toxic GOF, which resists simple gene supplementation. These cases require a *silence-and-replace* approach. CRISPR/Cas9-based vectors have been employed to suppress endogenous mutant *BEST1* expression (non-selective or allele-specific), coupled with exogenous WT gene delivery [76, 139]. This dual strategy successfully restored normal chloride channel function in iPSC-RPEs carrying three different GOF pathogenic variants, establishing feasibility for broad application across bestrophinopathy variants [74, 76, 135].

## Functional assay for AAV-*BEST1* in cell lines

A key advancement supporting clinical translation of *BEST1* gene therapy is the development of a functional in vitro assay using HEK293 cells. This assay measures chloride conductance via whole-cell patch-clamp to confirm the efficacy of AAV-mediated *BEST1* delivery. Protein expression correlates with functional activity, and the inclusion of the WPRE (Woodchuck Hepatitis Virus Post-transcriptional Regulatory Element) enhances both. This reproducible and GMP-compatible assay serves as a critical potency and quality control tool for future clinical trials [58].

## Pharmacological therapy for bestrophinopathies

While genomic therapies have dominated recent research, pharmacological approaches remain valid and promising. Valproic acid, in combination with rapamycin, has been shown to modulate proteolytic machinery and rescue photoreceptor outer segment processing defects in patient-derived iPSC-RPE, while also reducing autofluorescent material and delaying disease progression in canine models of ARB [140]. Additionally, molecular chaperones and proteasome inhibitors such as bortezomib and 4-phenylbutyrate (4PBA) have demonstrated the ability to correct trafficking of mutant *BEST1* and restore channel activity in cell models. 4PBA and its analogue 2-naphthoxyacetic acid have further shown efficacy in enhancing *BEST1* expression and chloride conductance in both patient-derived iPSC-RPE and HEK293 cells [66]. Despite their potential, many pharmacological agents require high concentrations and exhibit pathogenic variant-specific variability, highlighting the need for more tailored strategies. Table 1 summarises clinical trials targeting the bestrophinopathies. See also Supplementary Material Table S2 for ADVIRC clinical trials.

**Table 1:** Summary of clinical trials targeting *BEST1* pathogenic variants. *Source* www.clinicaltrials.gov

Gene / Drug Strategy	Vector/ Delivery	Clinical Condition	Inheritance / Mechanism	Disease Model / Population	Outcome
Gene augmentation [62]	Baculovirus vector	ARB	Autosomal recessive	iPSC-RPE cells	Rescue of Ca^2^⁺-dependent Cl⁻ current (whole-cell patch clamp)
Gene augmentation [113]	AAV2 vector	ARB (canine model)	Autosomal recessive	Canine BEST1 disease model	Reversal of subretinal detachment and micro detachment; correction of PR/RPE interface
Gene augmentation [116]	Lentiviral vector	ARB	Autosomal recessive	iPSC-RPE cells	Increased BEST1 protein; restoration of Ca^2^⁺-activated Cl⁻ current; improved RPE function (rhodopsin degradation)
Gene augmentation [116]	Lentiviral vector	BVMD	Autosomal dominant	iPSC-RPE cells	Increased BEST1 protein; restoration of Ca^2^⁺-activated Cl⁻ current; improved RPE function
CRISPR/Cas9-mediated knock-out of mutant allele [116]	Lentiviral vector (Cas9 + sgRNA)	BVMD	Autosomal dominant	iPSC-RPE cells	Increased BEST1 protein; restoration of Ca^2^⁺-activated Cl⁻ current
Gene augmentation [50]	AAV2 vector / Baculovirus vector	BVMD	Autosomal dominant (loss-of-function)	iPSC-RPE	Rescue of Ca^2^⁺-dependent Cl⁻ current (whole-cell patch clamp)
CRISPR/dCas9-mediated knock-down of both alleles + gene augmentation [67]	(i) Baculovirus expressing dCas9-KRAB-MeCP2; (ii) Baculovirus expressing WT BEST1	*BEST1*-related diseases	Autosomal dominant (gain-of-function)	hPSC-RPE H1-iCas9 cells	Rescue of Ca^2^⁺-dependent Cl⁻ current
Gene augmentation (OPGx-BEST1) NCT07185256	AAV-based vector; single subretinal injection	BVMD and ARB (BIRD-1 Phase 1/2 trial, NCT07185256)	BEST1-related IRD (AD BVMD and AR ARB)	Adult patients (≥ 18 years) with BVMD or ARB due to BEST1 mutations	Ongoing multi-center, adaptive, open-label, dose-exploring Phase 1b/2a trial; primary endpoints: safety and tolerability over 5 years; secondary/exploratory endpoints: dose finding and preliminary efficacy (visual function, retinal structure)
Natural history / registry (non-interventional, trial-enabling) NCT05809635	Standard clinical imaging and functional testing	BVMD	*BEST1* mutations (various AD/AR)	Children and adults with BEST1 VMD	Prospective natural history and genotype–phenotype correlation to support endpoint selection and trial design; no therapeutic intervention

## The clinical electrooculogram

The clinical EOG was described by Arden et al. (1962) [141]who proposed that the LP:DT_ratio_ could be used as a test of retinal function based on previous studies that investigated the interactions of light and the standing potential in retinal disease [141–143]. The standard model for the light-rise is based on an interaction between the rods and RPE and includes a ‘light-rise’ substance that is released from the dark adapted rods which either binds to or permeates the RPE to increase the basolateral membrane conductance which results in a positive shift in the recorded standing potential of the eye [50, 52, 75, 141]. The clinical EOG has been standardized since 1993 [144] when the protocol of dark adaptation followed by light adaptation to record the changes in the standing potential of the eye during saccadic eye movements at minute intervals was established.

The standing potential is the corneo-fundal potential [145] that has a positive value by convention and is generated principally across the RPE [146]. The clinical features of the EOG include the fall in the standing potential during a period of dark adaptation forming a ‘dark trough’ (DT). The DT minimum occurs ~ 10–15 min during the 15-min dark adaptation period. Following a 15-min period of white light adaptation of 100 cd/m^2^ background luminance the standing potential increases (termed the ‘light-rise’) to reach a maximum ‘light peak’ (LP) at approximately 7–12 min before declining again [1]. Clinically the main measure used is the ratio of the LP:DT formally known as the Arden Index or Arden ratio and from a meta-analysis has a mean value of 2.35 (95% CI 2.28–2.42) in dilated subjects and a mean value of 2.37 (95% CI 2.28–2.45) in nondilated subjects [147].

## Adapted clinical electrooculogram protocols

According to the ISCEV standard the clinical EOG involves recording of the standing potential of the eye through eye movements and alternating fixation during a 15-min dark-adaptation period, followed by an additional 15 min of measurement in light-adapted conditions [1, 52, 141]. EOG recording procedure is simple, but it is prone to artefacts, especially due to irregular eye movements or changes of posture during the examination. Such difficulties are frequently attributed to the prolonged and monotonous nature of the examination, which can lead to diminished concentration, discomfort, and fatigue of the patients. Furthermore, in pediatric and disabled patient populations, maintaining stable eye movements throughout the full 30-min recording session may not always be feasible. Thus, shorter-duration tests may be beneficial as they may be better tolerated in certain clinical circumstances while reducing the testing burden on the patient.

Türksever et al. (2015) [49] were the first to demonstrate the feasibility of short-duration EOG recording. Their recording protocol comprised a total dark phase duration of 10 min, consisting of 6 min of pre-adaptation to the dark, followed by 4 min of alternating fixation. A light phase was established with a total duration of 14 min, which included 4 min of light adaptation followed by 10 min of alternating fixation. Their work considered previously identified physiological factors that influenced the amplitude of the DT and LP [141–143]. As such, the recorded characteristics of this short-duration EOG protocol closely resembled those of the ISCEV standard EOG recordings. The median values of LP:DT_ratio_ were 2.25 and 2.4 (according to age-group), with the respective lower confidence limit between 1.60 and 1.77, which is equal to the values specified in the current ISCEV Standard (typically ranging from 1.7 to 4.3) [1].

**Fig. 4:**
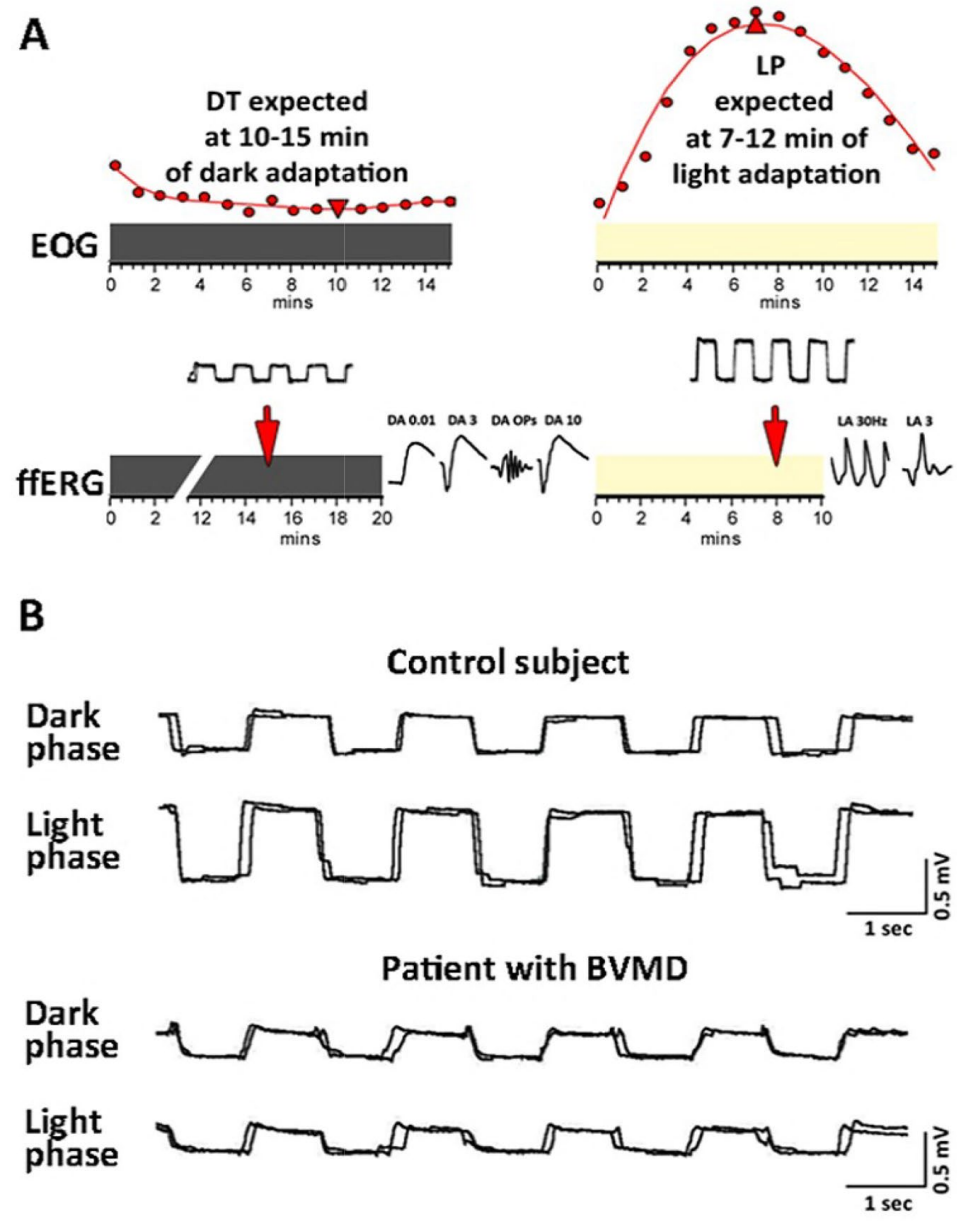
**A** The concept of integrating screening electrooculography (EOG) into the ffERG examination, as presented in the study by Djukanovic et al. (2024) [48]. The dark line represents dark adaptation, and the light line represents light adaptation. DT -dark trough, LP—light peak. **B** The recordings obtained from screening EOG during dark and light adaptation. In the control subject, a notable increase in the standing potential was observed during the light phase, while in a patient diagnosed with Best Vitelliform Macular Dystrophy (BVMD), the standing potential did not increase during light adaptation

The clinical EOG is usually conducted as only one of a broader set of tests within routine electrophysiological assessments. Simultaneous recording of ffERG is often necessary, to ensure accurate interpretation of EOG findings by excluding potential rod dysfunction. The need to perform both tests not only prolongs the overall duration of the electrophysiological assessment but also exacerbates patient discomfort. Djukanović et al. (2024) [48] attempted to further shorten the duration of testing by combining both examinations into one. Their protocol was based on the understanding that both EOG and ffERG require periods of dark and light adaptation. The primary output of the EOG test, specifically the LP:DT_ratio_, corresponds to critical time points during the dark and light adaptation phases when DT and LP are expected. If these two time points are evaluated during the dark and light adaptation periods within ffERG testing, then simultaneous EOG testing is possible (see Fig. 4). The EOG protocol was called a screening EOG because it consisted of only two recordings of the standing potential in the dark, and two in the light, but demonstrated clinical results that were comparable to the standard ISCEV EOG, with similar sensitivity and specificity in identifying RPE dysfunction in patients diagnosed with BVMD [48].

However, the screening EOG protocol according to Djukanovic et al. (2024) [48] gave slightly lower values in the control population compared to the standard EOG (1.98 ± 0.33 with screening EOG vs. 2.67 ± 0.61 with standard protocol) and lower than those reported by Türksever et al. (2015) [49]. This discrepancy may have arisen due to several factors, the first was that the simultaneous measurement of ffERG responses, performed by Djukanovic et al. (2024) [48]may have influenced the result of the screening EOG. During the dark phase of their protocol, the retina was partially stimulated by relatively intense flashes of dark-adapted ERG recordings. Besides, background luminance for the light phase of the screening EOG was aligned with the ffERG, which, however, requires lower luminance than those recommended for standard EOG (30 cd/m^2^ for ffERG vs. 100 cd/m^2^ for EOG). Furthermore, Djukanovic et al. (2024) [48] measured the standing potential in one-time point only, which may have contributed to less precise assessment of significant changes, as individual variations in the appearance of the LP and DT time points are likely to occur. Further improvements of integrated methodology could be obtained if the protocol implemented by Türksever et al. (2015) [49] (dark phase: 6 min of adaptation, 4 min of alternating fixation; light phase: 4 min of light adaptation, 10 min of alternating fixation) would be integrated into the ffERG investigation. Such a protocol could maintain the precision of standard methodology while reducing the testing burden on the patient, as attempted by Djukanovic et al. (2024) [48].

## Fast oscillation

One additional test, that is not commonly performed, is the Fast Oscillation (FO) of the ocular standing potential. In the FO the standing potential reduces during the light phase and increases in amplitude during the dark phase that originates in the RPE [148]. In contrast to the clinical EOG the dark and light phase intervals are 1 min in duration and typically 4–5 cycles are recorded taking approximately 10 min to complete the test. The fall in the standing potential during the light phase is due to the delayed basolateral membrane hyperpolarization [149–151] caused by a fall in sub retinal potassium following light absorption by the photoreceptors and inhibition of the outward potassium current mainly from the inner segments [152, 153]. The reduction in sub-retinal potassium slows the apical NaK2Cl cotransporter reducing the intracellular chloride concentration and basolateral chloride conductance resulting in the hyperpolarization of the basolateral membrane which causes a fall in the standing potential that reaches a minimum approximately 30 s after light onset [154]. The FO is not routinely recorded clinically but has been shown to have a larger amplitude following acute increase in blood glucose levels [155] and reduced in diabetes [156]. The FO is unaffected in BVMD [157] but may serve as a functional test for homeostasis of fluid in the sub-retinal; space given its dependence on NaK2Cl co-transporter. For recording the FO see the ISCEV EOG standard [1] and Mergaerts et al. (2001) [158]. The FO is also demanding on the patient with continuous saccades required to be performed during the light and dark cycles over 10–12 min. Modifications to the recommended protocol to reduce the number of cycles and/or the timing of the saccadic recording intervals to the anticipated peaks and troughs may lead to the FO being utilized more frequently clinically in the future.

## Discussion

The bestrophinopathies represent a clinically and genetically heterogeneous group of inherited retinal disorders that can be assessed using the EOG [6, 8]. This companion review to the mechanism of the EOG, provides an overview of disease-causing variants in *BEST1* can give rise to a spectrum of phenotypes ranging from the classic vitelliform lesions of BVMD to peripheral retinal changes seen in ADVRIC, and the multifocal presentations characteristic of ARB [7, 9, 42]. One unifying finding across these conditions is the abnormal EOG light-rise, reflecting widespread RPE dysfunction that may not correlate with the clinical appearance [39, 116]. Whilst genotype–phenotype correlations remain complex [43–45], the common EOG abnormalities provide a functional clinical marker that points to the role of bestrophin-1 in maintaining RPE physiology through regulation of intracellular calcium [11, 12].

Some potential therapeutic approaches for the bestrophinopathies, include gene therapies in canine models using AAV vectors that have restored *BEST1* function with structural and functional changes lasting several years [63, 135]. In addition, patient-derived iPSC-RPE models have enabled the identification and testing of potential therapeutic interventions by providing disease-causing variant-specific platforms for testing both gene replacement and pharmacological interventions [66, 69, 71–73, 76]. The distinction between LOF and GOF pathogenic variants has informed the development of tailored therapeutic strategies, with simple gene augmentation proving effective for most variants, while DN variants require more sophisticated "silence-and-replace" approaches using CRISPR-Cas9 technology [76, 139]. In addition, small molecule therapeutics, including 4-phenylbutyrate and tadalafil, offers clinical interventions to further support restoration of *BEST1* function [64, 66].

The clinical utility of the EOG in diagnosing bestrophinopathies is established but extending the current clinical utility of the EOG could be enhanced if the testing time was reduced from the current recommended 30 min [1]. The suggestion of using alternative shortened EOG protocols, even if as a screening instrument, represent a step towards making the EOG a more accessible and patient-friendly [48, 49]. The successful implementation of 10–14-min protocols that reduce test time [49] as well as integrating EOG recording within the ISCEV standard ERG protocol [48], may support wider use of the EOG as part of routine electrophysiological assessment during standard electrophysiological assessment.

The use of the EOG may increase to monitor outcomes for novel gene or therapeutic therapies that target the bestrophinopathies [62] in conjunction with structural imaging of the RPE [104]. From the first description of the clinical EOG by Arden et al. (1962) [141] that was standardized in the first ISCEV EOG standard [144] there have been no major amendments to the clinical recording. Now, strategies to reduce testing time of the EOG [48, 49] may provide an impetus to develop a shortened EOG test protocol. Modifications to the wavelength using a short wavelength 30 cd/m^2^ [159] stimulus may also offer a new testing strategy to be devised and validated [160]. If as the companion paper suggests that the light-rise reflects the availability of ATP and the metabolic health of the RPE then the EOG may find additional uses in evaluating the functional integrity of the EOG in a short and patient friendly protocol.

The last major review of the electrooculogram was by Arden and Constable (2006) [52] which predated the understanding of bestrophin’s role as a intracellular Ca^2+^ regulator and the identification of anoctamin ion channels in the RPE that is discussed in the companion paper. With the EOG and FO being the only functional electrophysiological tests with which to identify central RPE pathology and the development of therapeutics targeting the RPE, these clinical test protocols could be revised to improve patient comfort, clinical utility and improved service delivery [161]. The intention of this and the companion paper is to provide an update on the EOG mechanism and suggest a central role for ATP in the generation of the light-rise. In addition, emphasising the need to further test and refine the proposed shortened [49], screening [48] and short wavelength at 30 cd/m^2^ [159, 160] stimuli in clinical populations to develop a modified EOG protocol.

## Supplementary Information

Below is the link to the electronic supplementary material.

Supplementary file1 (PDF 240 KB)

## Data Availability

No datasets were generated or analysed during the current study.
