# Supplementary material for: Review of the clinical electrooculogram - Part 2: the bestrophinopathies and modified protocols
Source: Doc Ophthalmol. 2026 Mar 8;152(3):287–307. doi: 10.1007/s10633-026-10093-y (PMC13194349; doi:10.1007/s10633-026-10093-y)
Supplement: Supplementary file 1 — Supplementary file1 (PDF 240 KB) [file 10633_2026_10093_MOESM1_ESM.pdf]

Supplementary Material

Clinical applications of the electrooculogram: The Bestrophinopathies and Modified Protocols: Part 2

Srikanta Kumar Padhy, Maja Sustar Habjan, Paul A. Constable

Table S1 summarises the reported maximal and minima values for the $LP:DT_{ratio}$ in published cases with the reported gene associated with the pathogenic variant. Publicatuiosn in which the $LP:DT_{ratio}$ was not reported have ben excluded but provide additional phenotypic descriptions and include Wang et al (2022) [1], Jaouni et al (2012) [2], Laich et al (2025) [3] and Polosa et al (2025) [4].

Table S1. $LP:DT_{ratio}$, reported in various types of bestrophinopathies

Bestrophinopathy	Gene	$LP:DT_{ratio}$	Reference
BVMD	*BEST1*	0,83 to 1,22	Shah et al (2020) [5]
		0,95 to 1,76	Meunier et al (2011) [6]
		1,29 to 1,32	Gupta et al (2024) [7]
		1,00 to 2,20	Schatz et al (2006) [8]
		1,10 to 1,50	Sohn et al (2006) [9]
		absent or reduced	Arora et al (2016) [10]
		reduced	Wong et al (2010) [11]
		reduced in 5 /12 eyes (0,98 to 3,10)	Querques et al (2011) [12]
		reduced	Lima de Carvalho et al (2019) [13]
		1,10 to 1,80	Low et al (2011) [14]
		1,13 to 1,90	Duncker et al (2014) [15]
		1,69 to 1,95	Singuri et al (2025) [16]
		1,66 to 1,93	Tatemoto et al (2025) [17]
		0,55 mean n=45 subjects	Bitner et al (2012) [18]
		1.50	Vincent et al (2011) [19]
	PRPH2	normal in most cases	Meunier et al (2014) [20]
	IMPG1	1,25 to 2,82	Manes et al (2013) [21]
	not defined	1,14 ± 0,10	Theischen et al (1997) [22]
		1,14 to 1,50	Ratra and Karra (2025) [23]
ARB	*BEST1*	0,93 to 1,53	Shah et al (2020) [5]
		reduced	Tekin et al (2024) [24]
		severely reduced to absent	Burgess et al (2008) [25]
		1,01 to 1,89	Khojasteh et al (2021) [26]
		1,00 to 1,25	Casalino et al (2021) [27]
		1,02 to 1,80	Li et al (2024) [28]
		1,00	Lee et al (2015) [29]
		1,00 to 1,10	Pomares et al (2012) [30]
		~1,10 Both eyes	Johnson et al (2015) [31]
ADVIRC	*BEST1*	reduced	Mainguy et al (2024) [32]
	not defined	1,30 to 1,70	Wöster and Roider (2018) [33]
AVMD	*BEST1*	reduced in most cases	Meunier et al (2014) [20]
		1,49 to >1,65	Renner et al (2004) [34]
	PRPH2	<1,50	Meunier et al (2011) [6]
	IMPG1	normal in most cases	Meunier et al (2014) [20]
		2,20 to 3,04	Vázquez-Domínguez et al (2022) [35]
	IMPG2	normal in most cases	Meunier et al (2014) [20]
	not defined	1,70	Jaffe and Schatz (1988) [36]
		1,30 to 1,90	Dubovy et al (2000) [37]
		1,38 to 2,62	Saito et al (2003) [38]
		1,63 ± 0,27	Theischen et al (1997) [22]

Table S2 summarises clinical trials for the Bestrophinopathies.

Table S2

cDNA (NM_004183.4)	Protein change	Exon	Zygosity	Inheritance	Notes on phenotype / evidence
c.248G>A	p.Gly83Asp	4	Heterozygous	AD	Peripheral chorioretinal degeneration with ADVIRC phenotype; first described as ADVIRC mutation in proband with typical peripheral band and anterior segment changes [38].
c.256G>A	p.Val86Met	4	Heterozygous	AD	Progressive peripheral retinal degeneration with hyperpigmented band, angle-closure glaucoma, microcornea, cataracts, iris dysgenesis, optic nerve dysplasia, reduced or extinguished ERG, borderline EOG, and visual acuity ranging from normal to no light perception [39].
c.704T>C	p.Val235Ala	6	Heterozygous	AD	ADVIRC family with iPSC-RPE studies showing *BEST1* mislocalisation; previously thought to alter splicing enhancer [40].
c.707G>A	p.Val235Ala	6	Heterozygous	AD	Exon-6 ADVIRC mutations such as c.704T>C and c.707G>A disrupt an exonic splice enhancer, leading to ESE-dependent exon skipping, increased ASF/SF2 binding, and production of shortened *BEST1* isoforms, distinguishing their mechanism from nearby mutations causing BVMD [41].
c.707A>G	p.Tyr236Cys	6	Heterozygous	AD	ADVIRC with variable expressivity; later functional work shows increased open probability of *BEST1* channel [42].
c.715G>A	p.Val239Met	7	Heterozygous	AD	The c.715G>A (p.Val239Met) *BEST1* variant causes a variable ADVIRC phenotype, ranging from classic slowly progressive peripheral vitreoretinal degeneration to severe early-onset retinal dystrophy. Affected individuals typically demonstrate circumferential peripheral retinal hyperpigmentation, progressive retinal dysfunction, visual field constriction, and reduced ffERG amplitudes-with photopic losses exceeding scotopic decline [43].
c.830C>T	p.Thr277Met	7	Heterozygous	AD	The c.830C>T (p.T277M) *BEST1* variant is associated with an atypical ADVIRC phenotype characterized by progressive visual decline, confluent mid-peripheral pigmentary chorioretinal atrophy with macular sparing, peripheral retinal thinning and structural disorganization on OCT, and absence of anterior segment abnormalities. This presentation demonstrates more posterior retinal involvement than typically observed in ADVIRC [44].
c.1712T>C	p.Met571Thr	10–11	Heterozygous	AD	The p.Met571Thr *BEST1* variant has been associated with an ADVIRC phenotype characterized by a distinct annular peripheral hyperpigmented band, preserved macular structure and function, and progressive peripheral retinal degeneration. A unique clinical feature reported with this variant is the development of subretinal hemorrhage (SRH) resembling peripheral exudative hemorrhagic chorioretinopathy, which improved following vitrectomy. Electroretinography revealed reduced photopic and scotopic responses, consistent with diffuse retinal dysfunction, while EOG measurements demonstrated preserved or borderline function. Unlike many typical ADVIRC cases, no anterior segment abnormalities or family history were present, suggesting a possible sporadic presentation or incomplete penetrance [45].<br>But its pathogenicity remains uncertain given contradictory in-silico predictions, lack of segregation, and low evolutionary conservation. Mano et al (2018) [42] argue that despite limited in-silico support, the c.1712T>C (p.Met571Thr) variant remains a plausible pathogenic cause of ADVIRC based on CLIA-certified laboratory classification, possible functional impact via altered phosphorylation, and a clinically consistent phenotype, urging caution in dismissing rare variants solely through computational prediction.

References

1. Wang Y, Jiang Y, Li X et al (2022) Genetic and clinical features of *BEST1*-associated retinopathy based on 59 Chinese families and database comparisons. Exp Eye Res 223:109217. https://doi.org/10.1016/j.exer.2022.109217
2. Jaouni T, Averbukh E, Burstyn-Cohen T, Grunin M, Banin E, Sharon D, Chowers I (2012). Association of pattern dystrophy with an *HTRA1* single-nucleotide polymorphism. Arch Ophthalmol 130:987-991. https://doi.org/10.1001/archophthalmol.2012.1483
3. Laich Y, Georgiou M, Fujinami K, Daich Varela M, Fujinami-Yokokawa Y, Hashem SA, de Guimaraes, TAC, Mahroo OA, Webster AR, Michaelides M (2025) Best Vitelliform Macular Dystrophy natural history study report 2: Fundus autofluorescence and OCT. Ophthalmol Retina 9:899-907. https://doi.org/10.1016/j.oret.2025.03.004
4. Polosa A, Lu M, Dorfman A L, Masis-Solano M, Costantino S, Qian CX (2025) Characterization of functional and structural impairments in best vitelliform macular dystrophy using visual electrophysiology and optical coherence tomography in pediatric and adult patients. Doc Ophthalmol 10.1007/s10633-025-10064-9. https://doi.org/10.1007/s10633-025-10064-9
5. Shah M, Broadgate S, Shanks M, et al (2020) Association of clinical and genetic heterogeneity with *BEST1* sequence variations. JAMA Ophthalmol 138:544-551. https://doi.org/10.1001/jamaophthalmol.2020.0666
6. Meunier I, Sénéchal A, Dhaenens CM, et al (2011) Systematic screening of BEST1 and PRPH2 in juvenile and adult vitelliform macular dystrophies: a rationale for molecular analysis. Ophthalmology 118:1130-1136. https://doi.org/10.1016/j.ophtha.2010.10.010
7. Gupta V, Chawla R, Kumar V (2024) Bull's-eye maculopathy in Best Vitelliform Dystrophy. Ophthalmol Retina 8:e51. https://doi.org/10.1016/j.oret.2024.05.007
8. Schatz P, Klar J, Andréasson S, Ponjavic V, Dahl N (2006) Variant phenotype of Best vitelliform macular dystrophy associated with compound heterozygous mutations in *VMD2*. Ophthalmic Genet 27:51-56. https://doi.org/10.1080/13816810600677990
9. Sohn EH, Francis PJ, Duncan JL et al (2009) Phenotypic variability due to a novel Glu292Lys variation in exon 8 of the BEST1 gene causing best macular dystrophy. Arch Ophthalmol 127:913-920. https://doi.org/10.1001/archophthalmol.2009.148
10. Arora R, Khan K, Kasilian ML et al (2016) Unilateral *BEST1*-associated retinopathy. Am J Ophthalmol 169:24-32. https://doi.org/10.1016/j.ajo.2016.05.024
11. Wong RL, Hou P, Choy KW et al (2010) Novel and homozygous BEST1 mutations in Chinese patients with Best vitelliform macular dystrophy. Retina 30:820-827. https://doi.org/10.1097/IAE.0b013e3181c700c1
12. Querques G, Zerbib J, Santacroce R et al The spectrum of subclinical Best vitelliform macular dystrophy in subjects with mutations in *BEST1* gene. Invest Ophthalmol Vis Sci 52:4678-4684. https://doi.org/10.1167/iovs.10-6500
13. Lima de Carvalho JR Jr, Paavo M, Chen L, Chiang J, Tsang SH, Sparrow JR (2019) Multimodal imaging in Best Vitelliform Macular Dystrophy. Invest Ophthalmol Vis Sci 60:2012-2022. https://doi.org/10.1167/iovs.19-26571
14. Low S, Davidson AE, Holder GE et al Autosomal dominant Best disease with an unusual electrooculographic light rise and risk of angle-closure glaucoma: a clinical and molecular genetic study. Mol Vis 2011:2272-2282.
15. Duncker T, Greenberg JP, Ramachandran R et al (2014) Quantitative fundus autofluorescence and optical coherence tomography in best vitelliform macular dystrophy. Invest Ophthalmol Vis Sci 55:1471-1482. https://doi.org/10.1167/iovs.13-13834
16. Singuri S, DeBenedictis MJ, Traboulsi EI, Yuan A, Schur RM (2025) *BEST1* variant associated with an atypical macular and peripheral retinal phenotype. Retin Cases Brief Rep 19:129-134. https://doi.org/10.1097/ICB.0000000000001520
17. Tatemoto Y, Hayashi T, Mizobuchi K, Den S, Nakano T (2025) Best vitelliform macular dystrophy caused by a *BEST1* p.(Ser246Asn) variant coexisting with diabetic retinopathy. Doc Ophthalmol 10.1007/s10633-025-10056-9. https://doi.org/10.1007/s10633-025-10056-9
18. Bitner H, Schatz P, Mizrahi-Meissonnier L, Sharon D, Rosenberg T (2012). Frequency, genotype, and clinical spectrum of best vitelliform macular dystrophy: data from a national center in Denmark. Am J Ophthalmol 154:403-412.e4. https://doi.org/10.1016/j.ajo.2012.02.036
19. Vincent A, McAlister C, Vandenhoven C, Héon E (2011) BEST1-related autosomal dominant vitreoretinochoroidopathy: a degenerative disease with a range of developmental ocular anomalies. Eye 25:113-118. https://doi.org/10.1038/eye.2010.165
20. Meunier I, Manes G, Bocquet B, Marquette V, Baudoin C, Puech B, Defoort-Dhellemmes S, Audo I, Verdet R, Arndt C, Zanlonghi X, Le Meur G, Dhaenens CM, Hamel CP (2014) Frequency and clinical pattern of vitelliform macular dystrophy caused by mutations of interphotoreceptor matrix *IMPG1* and *IMPG2* genes. Ophthalmology 121:2406-2414. https://doi.org/10.1016/j.ophtha.2014.06.028
21. Manes G, Meunier I, Avila-Fernández A, Banfi S, Le Meur G, Zanlonghi X et al (2013). Mutations in *IMPG1* cause vitelliform macular dystrophies. Am J Hum Genet 93:571-578. https://doi.org/10.1016/j.ajhg.2013.07.018
22. Theischen M, Schilling H, Steinhorst UH (1997) EOG in adult vitelliform macular degeneration, butterfly-shaped pattern dystrophy and Best disease. Ophthalmologe 94:230-233. https://doi.org/10.1007/s003470050107
23. Ratra D, Karra A (2025) Unilateral best vitelliform macular dystrophy- a case series. Doc Ophthalmol 150:111-116. https://doi.org/10.1007/s10633-025-10008-3
24. Tekin K, Dulger SC, Horozoglu Ceran T, Inanc M, Ozdal PC, Teke MY (2024) Multimodal imaging and genetic characteristics of autosomal recessive bestrophinopathy. J Fr Ophtalmol 47:104097. https://doi.org/10.1016/j.jfo.2024.104097

25. Burgess R, Millar ID, Leroy BP et al (2008) Biallelic mutation of *BEST1* causes a distinct retinopathy in humans. Am J Hum Genet 82:19-31. https://doi.org/10.1016/j.ajhg.2007.08.004
26. Khojasteh H, Azarmina M, Ebrahimiadib N et al (2021) Autosomal Recessive Bestrophinopathy: clinical and genetic characteristics of twenty-four cases. J Ophthalmol 2021:6674290. https://doi.org/10.1155/2021/6674290
27. Casalino G, Khan KN, Armengol M et al (2021) Autosomal Recessive Bestrophinopathy: Clinical features, natural history, and genetic findings in preparation for clinical trials. Ophthalmology 128:706-718. https://doi.org/10.1016/j.ophtha.2020.10.006
28. Li JX, Meng LR, Hou BK et al (2024) Detection of novel *BEST1* variations in Autosomal Recessive Bestrophinopathy using third-generation sequencing. Curr Med Sci 44:419-425. https://doi.org/10.1007/s11596-024-2865-3
29. Lee CS, Jun I, Choi SI et al (2015) A novel *BEST1* Mutation in Autosomal Recessive Bestrophinopathy. Invest Ophthalmol Vis Sci 56:8141-8150. https://doi.org/10.1167/iovs.15-18168
30. Pomares E, Burés-Jelstrup A, Ruiz-Nogales S, Corcóstegui B, González-Duarte R, Navarro R (2012) Nonsense-mediated decay as the molecular cause for autosomal recessive bestrophinopathy in two unrelated families. Invest Ophthalmol Vis Sci 53:532-537. https://doi.org/10.1167/iovs.11-7964
31. Johnson AA, Bachman LA, Gilles BJ et al (2015) Autosomal Recessive Bestrophinopathy is not associated with the loss of bestrophin-1 anion channel function in a patient with a novel *BEST1* mutation. Invest Ophthalmol Vis Sci 56:4619-4630. https://doi.org/10.1167/iovs.15-16910
32. Mainguy A, Dhaenens CM, Poncet A et al Variable expressivity of the autosomal dominant vitreoretinochoroidopathy (ADVIRC) phenotype associated with a novel variant in *BEST1*. Ophthalmic Genet 45:470-475. https://doi.org/10.1080/13816810.2024.2368797
33. Wöster L, Roider J (2018) Long-term changes in autosomal dominant vitreoretinochoroidopathy (ADVIRC). Graefes Arch Clin Exp Ophthalmol 256:441-442. https://doi.org/10.1007/s00417-017-3810-y
34. Renner AB, Tillack H, Kraus H et al (2004) Morphology and functional characteristics in adult vitelliform macular dystrophy. Retina 24:929-939. https://doi.org/10.1097/00006982-200412000-00014
35. Vázquez-Domínguez I, Li, CHZ, Fadaie Z, Haer-Wigman L, Cremers FPM, Garanto A, Hoyng CB, Roosing S (2022) Identification of a complex allele in *IMPG2* as a cause of adult-onset vitelliform macular dystrophy. Invest Ophthalmol Vis Sci 63:27. https://doi.org/10.1167/iovs.63.5.27
36. Jaffe GJ, Schatz H (1988) Histopathologic features of adult-onset foveomacular pigment epithelial dystrophy. Arch Ophthalmol 106:958-960. https://doi.org/10.1001/archopht.1988.01060140104034
37. Dubovy SR, Hairston RJ, Schatz H, Schachat AP, Bressler NM, Finkelstein D, Green WR (2000) Adult-onset foveomacular pigment epithelial dystrophy: clinicopathologic correlation of three cases. Retina 20:638-649. https://doi.org/10.1097/00006982-200011000-00009
38. Saito W, Yamamoto S, Hayashi M, Ogata K (2003) Morphological and functional analyses of adult onset vitelliform macular dystrophy. Br J Ophthalmol 87:758-762. https://doi.org/10.1136/bjo.87.6.758
39. Goldberg MF, Lee FL, Tso MO, Fishman GA (1989) Histopathologic study of autosomal dominant vitreoretinochoroidopathy. Peripheral annular pigmentary dystrophy of the retina. Ophthalmology 96:1736-1746. https://doi.org/10.1016/s0161-6420(89)32663-7
40. Vincent A, McAlister C, Vandenhoven C, Héon E (2011) BEST1-related autosomal dominant vitreoretinochoroidopathy: a degenerative disease with a range of developmental ocular anomalies. Eye 25:113-118. https://doi.org/10.1038/eye.2010.165
41. Carter DA, Smart MJ, Letton WV, Ramsden CM, Nommiste B, Chen LL et al (2016) Mislocalisation of *BEST1* in iPSC-derived retinal pigment epithelial cells from a family with autosomal dominant vitreoretinochoroidopathy (ADVIRC). Sci Rep 6:33792. https://doi.org/10.1038/srep33792
42. Burgess R, MacLaren RE, Davidson AE, Urquhart JE, Holder GE, Robson AG et al (2009). ADVIRC is caused by distinct mutations in *BEST1* that alter pre-mRNA splicing. J Med Genet 46:620-625. https://doi.org/10.1136/jmg.2008.059881
43. Yardley J, Leroy BP, Hart-Holden N, Lafaut BA, Loeys B, Messiaen LM et al (2004). Mutations of *VMD2* splicing regulators cause nanophthalmos and autosomal dominant vitreoretinochoroidopathy (ADVIRC). Invest Ophthalmol Vis Sci 45:3683-3689. https://doi.org/10.1167/iovs.04-0550
44. da Palma MM, Vargas ME, Burr A, Chen R, Pennesi ME, Weleber RG, Yang P (2021) Variable expressivity of *BEST1*-associated autosomal dominant vitreoretinochoroidopathy (ADVIRC) in a three-generation pedigree. BMJ open ophthalmology 6:e000813. https://doi.org/10.1136/bmjophth-2021-000813
45. Komro J, Skender S, Ross BX, Lin X (2022) Autosomal Dominant Vitreoretinochoroidopathy with a novel *BEST1* mutation and a review of reported mutations. Cureus 14:e32990. https://doi.org/10.7759/cureus.32990
46. Mano F, LoBue SA, Olsen TW, Marmorstein AD, Pulido JS (2018) A novel missense mutation in *BEST1* associated with an autosomal-dominant vitreoretinochoroidopathy (ADVIRC) phenotype. Ophthalmic Genet 39:749-753. https://doi.org/10.1080/13816810.2018.1520264
